# Validating a Major Quantitative Trait Locus and Predicting Candidate Genes Associated With Kernel Width Through QTL Mapping and RNA-Sequencing Technology Using Near-Isogenic Lines in Maize

**DOI:** 10.3389/fpls.2022.935654

**Published:** 2022-06-30

**Authors:** Yanming Zhao, Xiaojie Ma, Miaomiao Zhou, Junyan Wang, Guiying Wang, Chengfu Su

**Affiliations:** ^1^College of Agronomy, Qingdao Agricultural University, Qingdao, China; ^2^Shandong Provincial Key Laboratory of Dryland Farming Technology, Qingdao Agricultural University, Qingdao, China

**Keywords:** maize, NILs, QTL validation, kernel width, candidate genes

## Abstract

Kernel size is an important agronomic trait for grain yield in maize. The purpose of this study was to validate a major quantitative trait locus (QTL), *qKW-1*, which was identified in the F_2_ and F_2:3_ populations from a cross between the maize inbred lines SG5/SG7 and to predict candidate genes for kernel width (KW) in maize. A major QTL, *qKW-1*, was mapped in multiple environments in our previous study. To validate and fine map *qKW-1*, near-isogenic lines (NILs) with 469 individuals were developed by continuous backcrossing between SG5 as the donor parent and SG7 as the recurrent parent. Marker-assisted selection was conducted from the BC_2_F_1_ generation with simple sequence repeat (SSR) markers near *qKW-1*. A secondary linkage map with four markers, PLK12, PLK13, PLK15, and PLK17, was developed and used for mapping the *qKW-1* locus. Finally, *qKW-1* was mapped between the PLK12 and PLK13 intervals, with a distance of 2.23 cM to PLK12 and 0.04 cM to PLK13, a confidence interval of 5.3 cM and a phenotypic contribution rate of 23.8%. The QTL mapping result obtained was further validated by using selected overlapping recombinant chromosomes on the target segment of maize chromosome 3. Transcriptome analysis showed that a total of 12 out of 45 protein-coding genes differentially expressed between the two parents were detected in the identified *qKW-1* physical interval by blasting with the Zea_Mays_B73 v4 genome. GRMZM2G083176 encodes the Niemann–Pick disease type C, and GRMZM2G081719 encodes the nitrate transporter 1 (NRT1) protein. The two genes GRMZM2G083176 and GRMZM2G081719 were predicted to be candidate genes of *qKW-1.* Reverse transcription-polymerase chain reaction (RT-qPCR) validation was conducted, and the results provide further proof of the two candidate genes most likely responsible for *qKW-1*. The work will not only help to understand the genetic mechanisms of KW in maize but also lay a foundation for further cloning of promising loci.

## Introduction

Maize is one of the most important agricultural crops and serves as a food, animal feed, and industrial material (Li et al., 2017). Maize is very important in food security (Liu et al., 2014). Obtaining high grain yield is one of the most important goals for breeders in maize breeding. However, many yield traits are complex quantitative traits that are controlled by multiple genes. Kernel size traits, including kernel length, kernel width (KW), and kernel thickness, are considered important traits affecting yield (Doebley et al., 2006). As one of the most important yield components, KW has high heritability and is less affected by the environment (Veldboom and Lee, 1994, 1996; Peng et al., 2011). Therefore, analyzing the genetic basis of KW has theoretical guiding significance for the innovation of germplasm resources and the cultivation of high-yielding varieties in maize breeding.

Along with the rapid development of maize genomics and utilization of novel molecular markers, the study of quantitative trait locus (QTL) mapping related to kernel size traits has made remarkable progress in maize. QTLs controlling important agronomic traits in maize were detected by analyzing phenotypic value based on constructed genetic maps. Veldboom and Lee (1994) developed an F_2:3_ population that contains 150 lines derived from a cross between Mo17 and H99. The population was used for QTL mapping of maize kernel-related traits. Five QTLs controlling kernel length were detected on chromosomes 1, 3, 6, 7, and 8, whereas six QTLs controlling 100-kernel weight were detected on chromosomes 1, 3, 4, 5, and 8 (Veldboom and Lee, 1994). Li et al. (2009) used the important maize inbred lines Huangzao number 4 and Qi 319 as materials to construct a segregation population. A population containing 226 F_2:3_ lines was applied to detect QTLs conferring kernel-related traits. The results showed that 15 kernel-related QTLs were detected in the F_2:3_ population, and most of the kernel-related traits were significantly correlated with each other, whereas kernel thickness was negatively correlated with maize yield (Li et al., 2009). Peng et al. (2011) used the maize inbred line 319 cross Ye 478 and Huangzao number 4, respectively, to construct segregation populations, and two populations containing 230 and 235 lines, respectively, were developed and applied for QTL mapping for kernel-related traits in maize. The results showed that a major QTL for KW was detected with a phenotypic contribution rate greater than 10% (Peng et al., 2011). Li et al. (2010c) constructed a recombinant inbred line (RIL) population, F_2:3_ groups and BC_2_F_2_ groups by crossing two maize inbred lines with different kernel shapes, Dan 232 and N04. QTL mapping results showed that 5 important meta-QTLs were detected on chromosomes 1, 4, 5, 7, and 10 (Li et al., 2010c). Qin et al. (2015) developed an RIL population with 130 lines by crossing Huangzao number 4 and Mo17 for mapping QTLs conferring KW in maize. A total of 4 KW QTLs were identified with contribution rates of 6.01–15.91%, among which the physical interval for Qqkwid 10 decreased to 143.4–143.7 Mb (Qin et al., 2015). Li et al. (2016) selected 627 important maize inbred lines as materials and used correlation analysis and stepwise regression methods to explore the kernel characteristics of Chinese maize inbred line germplasm resources. The results showed that the contribution rate of KW to 100-grain volume is between 54 and 71% (Li et al., 2016). Many QTL mapping or fine mapping works for kernel size or kernel weight have been completed in recent years (Liu et al., 2014; Zhang et al., 2014; Chen et al., 2016). To date, more than 150 QTLs for kernel size or weight have been identified by using different maize populations (Gramene QTL database).

It is especially important that QTLs be validated and fine mapped for application in further marker-assisted breeding processes. Near-isogenic lines (NILs) are among the most widely accepted populations commonly used in QTL fine mapping. NILs have been successfully used in confirming and fine mapping QTLs in many species, such as rice (Lin et al., 2003; Li et al., 2004; Xie et al., 2006) and wheat (Xue et al., 2013; Zheng et al., 2015; Zhang et al., 2020). In maize, Nie et al. (2019) mapped a major QTL, *qkrnw4*, associated with kernel row number with an NIL. Gao et al. (2019) mapped *qLRI4* that conferred leaf rolling index by using NIL populations. Yang et al. (2018) mapped a major QTL, *qkc7.03*, to a 416.27 kb physical interval for kernel cracking with developed NILs.

Great achievements in QTL mapping or isolating underlying genes for kernel size have been made in many species, such as rice (Wan et al., 2006; Song et al., 2007; Li et al., 2011; Qiu et al., 2012; Kang et al., 2018), *Arabidopsis thaliana* (Xia et al., 2013; Du et al., 2014), soybean (Xu et al., 2011; Han et al., 2012), and wheat (Sun et al., 2009; Ramya et al., 2010). In particular, genes controlling rice kernel size or weight, such as *GS3* (Fan et al., 2006), *GS5* (Li et al., 2011), *qGL3* (Zhang et al., 2012), *GW2* (Song et al., 2007), *GW8* (Wang et al., 2012), *GS2* (Hu J. et al., 2015), and *qGW7/GL7* (Wang et al., 2015), have been successfully cloned. The study of identifying and cloning kernel size-related genes has lagged in maize. Both maize and rice are diploid monocotyledonous gramineous plants. The homologous gene in maize was obtained by blasting the rice genome: this gene most likely controls the same trait in the two species of maize and rice. To date, a large number of genes associated with maize kernels have been identified by using homology-based cloning technology. Li et al. (2010a) successfully cloned two genes, *ZmGW2*–*CHR4* and *ZmGW2*–*CHR5*, in a natural maize population by blasting the kernel-related gene *GW2* sequence in rice. The *GW2* gene in rice is a pleiotropic gene that correlates not only with KW but also kernel weight. Li et al. (2010b) successfully cloned the *Zm-GS3* gene in maize by blasting the sequence of the rice kernel-related gene *GW3.* Previous studies have shown that *GW3* is a pleiotropic gene related to kernel length and kernel weight in rice. After cloning and functional analysis of the *Zm-GS3* gene, it was found that the *Zm-GS3* gene is closely related to kernel development in maize. Liu et al. (2015) successfully cloned the *Zm-GS5* gene in maize by blasting the rice kernel shape-related gene *GS5* sequence. *Zm-GS5* is limited to a small segment on chromosome 3. Previous studies have used the BC_2_F_6_ population through multigeneration backcrossing and selfing of the ruminant grass and maize inbred line Mo17 for mapping kernel-related QTLs. A major QTL for kernel weight is limited to a segment of chromosome 3, which is consistent with the segment of the gene *Zm-GS5* found in the results of Liu et al. obtained in 2015 (Liu et al., 2015).

Based on previous studies, the purposes of this study were (1) to validate and fine map the identified major QTL *qKW-1* by using BC_4_F_2_ NILs and (2) to reveal differentially expressed genes (DEGs) between SG5 and SG7 by RNA-sequencing (RNA-seq) technology and predict candidate genes responsible for KW.

## Materials and Methods

### Near-Isogenic Lines Development and Phenotyping

**FIGURE 1:**
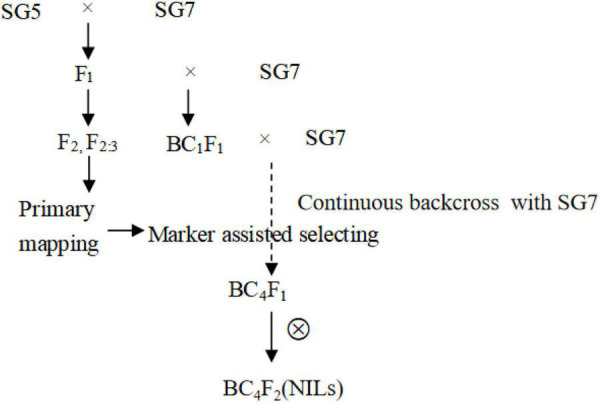
The development process of NILS based on primary mapping results in F_2_ population from the cross between *Zea mays* L. SG5 and *Zea mays* L. SG7. Polymorphic simple sequence repeat (SSR) markers between SG5 and SG7 was used for marker assisted selection from BC_2_F_1_ till BC_4_F_1_ generation. Heterozygous genotypes for *qKW-1* locus were selected by using polymorphic SSR markers in *qKW-1* region each generation.

Near-isogenic lines for the *qKW-1* locus were developed by using continuous backcrossing combined with the marker-assisted selection method (Figure 1). F_1_ hybrid seeds were obtained from a cross between *Zea mays* L. SG5 and *Zea mays* L. SG7 in the summer of 2018 in Qingdao, Shandong Province, China. F_1_ hybrid seeds were planted in winter 2018 in Sanya, China. BC_1_ seeds were obtained from the backcross between F_1_ (female parent) and *SG7* (recurrent parent). BC_2_ to BC_4_ generation materials were planted and obtained alternately in Sanya of Hainan Province and Qingdao of Shandong Province in China from 2019 to 2021. A total of 469 BC_4_F_2_ plants were then planted in Qingdao for phenotyping in the summer of 2021.

A total of 50 simple sequence repeat (SSR) molecular markers (Supplementary Table 1) within approximately 10 Mb of the physical interval in which *qKW-1* was located were selected for polymorphism screening. These SSR primers were synthesized by Shanghai Institutes for Biological Sciences according to the sequences published based on resequencing maize genome results (Xu et al., 2013). Polymorphic SSR markers between donor parent SG5 and recurrent parent SG7 were used for marker-assisted selection from the BC_2_F_1_ to BC_4_F_1_ generation. From the BC_2_F_1_ to BC_4_F_1_ generations, heterozygous genotypes for the *qKW-1* locus were selected by using polymorphic SSR markers in the *qKW-1* region of each generation. The BC_4_F_2_ population applied for QTL fine mapping was developed by selfing several BC_4_F_1_ individuals with heterozygous *qKW-1* loci.

The BC_4_F_2_ individuals were planted in the field within boundary rows by using a completely randomized design method. A single-row plot with row spacing of 50 cm was adopted, and each plot grew 15 plants with plant spacing of 35 cm. The KW trait was investigated after the corns were harvested and dried naturally. The phenotypic value of each BC_4_F_2_ individual was estimated by the mean value of 10 kernels randomly selected from the middle part of the ear.

### DNA Extraction and Genotyping

Young healthy leaves from the two parents, and each of the BC_4_F_2_ individuals were collected and frozen in liquid nitrogen and then transferred to an −80°C freezer. Genomic DNA from the BC_4_F_2_ population and parents was extracted following the manufacturer’s protocols with the Plant Genomic DNA Kit (TIANGEN, Beijing, China). DNA degradation and contamination were monitored on 1% agarose gels. DNA purity was checked using a NanoPhotometer^®^ spectrophotometer (IMPLEN, Westlake Village, CA, United States). The DNA concentration was measured using a Qubit^®^ DNA Assay Kit with a Qubit^®^ 2.0 Fluorometer (Life Technologies, CA, United States).

Polymorphic SSR markers in the objective heterozygous *qKW-1* segment were applied for polyacrylamide gel electrophoresis (PAGE) analysis. Recoding alleles for each SSR in each BC_4_F_2_ individual, heterozygous genotypes are coded as 1. Homozygous genotypes identical to SG5 were coded as 0, whereas homozygous genotypes identical to SG7 were coded as 2.

The optimal system was in a 10-μl reaction volume containing 5 μl 2 × 3G Taq Master Mix with Red Dye for PAGE (Vazyme Biotech Co., Ltd., Nanjing, Jiangsu, China), 10 μmol/L forward primer and 1 μl reverse primer, respectively, and 15–20 ng template DNA, with ultrapure grade water added to a total volume of 10 μl. The polymerase chain reactions (PCRs) were performed in BIO-RAD-T100 Thermal Cyclers with the following program: 95°C, 3 min; 95°C, 15 s; 60°C, 15 s, and 72°C, 1 min, followed by 34 additional cycles. PCR products were kept at 4°C and electrophoresed on an 8% native-PAGE gel. Gels were run in PAGE running buffer (1 × TBE) at 120 V for 3 h and then silver-stained. Recoding alleles for each SSR in each BC_4_F_2_ individual, heterozygous genotypes are coded as 1. Homozygous genotypes identical to SG5 were coded as 0, whereas homozygous genotypes identical to SG7 were coded as 2.

### Near-Isogenic Lines Genetic Background Assessment and Quantitative Trait Locus Validation

Five SSR markers were randomly selected from each of the other nine chromosomes except chromosome 3, in which the *qKW-1* locus was located. Forty-five SSR markers were applied for BC_4_F_2_ genetic background assessment. The secondary linkage map of the *qKW-1* segment was generated by JoinMap 3.0 software (Van Ooijen and Voorrips, 2001) with a logarithm of the odds (LOD) threshold of 10.0. QTL Cartographer v2.5 was applied for QTL mapping with the CIM method, walking speed 1 cM. The LOD score threshold was determined by the result of 1,000 permutations for the KW trait.

### Candidate Gene Prediction for *qKW-1* by RNA-Sequencing Technology

Grains of SG5 and SG7 were sampled on the 5th, 10th, and 15th days after selfing, with three biological replicates. All collected samples were immediately frozen in liquid nitrogen and then transferred to a −80°C environment before RNA extraction. We obtained 18 grain samples in total. All samples were sequenced on the Illumina NovaSeq platform. Raw reads in fastq format were first processed by in-house Perl scripts. Clean reads were then obtained after deleting reads containing adapter and poly-N sequences and removing reads of low quality from the raw data. In addition, the guanine–cytosine (GC) content, Q20 and Q30 of the clean reads were calculated. High-quality clean data were then used for further downstream analysis. The reference genome was downloaded directly from the genome website,^1^ and correlated files of gene annotation were also downloaded from the same website. Bowtie v2.2.3 was used to build the reference genome index, and TopHat v2.0.12 (Trapnell et al., 2013) was used to align paired-end clean reads to the reference genome. The number of reads mapped to each gene was counted by HTSeq v0.6.1. For each gene, the expected number of fragments per kilobase of transcript sequence per million base pairs (FPKM) was calculated by analyzing the gene length and reads mapped to the gene. FPKM is a widely accepted method currently used to evaluate levels of gene expression based on considering the sequencing depth effect and gene length of the read count simultaneously (Trapnell et al., 2010). The DEGSeq R package (1.20.0) was applied to analyze differential expression between the two conditions. The *p*-values adjusted by using the Benjamini and Hochberg method were used. The threshold of corrected *p*-value 0.005 and log 2 (fold change) of 1 (absolute value) was considered to represent significantly DEGs. More information about the methods for reference genome index construction, paired-end clean read alignment and count, FPKM calculation, and DEG analysis is provided in our previous study (Zhao and Su, 2019).

By analyzing DEGs between SG5 and SG7, the DEGs that were overlaid onto the physical interval of *qKW-1* were considered candidate genes for kernel size in maize. The detected DEGs were further annotated based on the Blast SwissProt database.

### Reverse Transcription-Polymerase Chain Reaction Analysis

Reverse transcription-polymerase chain reaction (RT-qPCR) experiments for candidate genes were performed to verify the reliability of the RNA-seq experimental data. RT-qPCR primers were designed by the Primer-BLAST^2^ online tool. Some basic principles for primer design are as follows: the length of the amplification product is between 50 and 150 bp, the primer length is between 18 and 24 nt, the difference between the *Tm* values of forward and reverse primers should not be too large (less than 3°C), the *Tm* values should be controlled between 58 and 64°C, and 60°C is the optimal temperature. The GC content in the 45–55% interval is the most suitable. To avoid complementation or hybridization between each pair of primers, the base at the 3′ end of the primer should preferably be G or C. The BLAST procedure is used to test the specificity of the primers. The Evo M-MLV RT-PCR Kit from Sangon Biotech (Shanghai) Co., Ltd. was used for reverse transcription of total RNA to synthesize the first strand of cDNA. The RT-PCR analysis was performed by a real-time fluorescence quantitative PCR instrument using the 2 × SYBR^®^ Green dye method. The actin gene was used as the internal reference gene, and 2^–△△Ct^ was used to analyze the results. The sequences of the primers are listed as follows: Actin (F: GTCCATGAGGCCACGTACAA; R: CCGGACCAGTTTCGTCATA), GRMZM2G083176 (F: GTTCA TGGCTAGATCCGGCA; R: ACGAAGTGAGATGACGACGG), and GRMZM2G081719 (F: CCGGCCACCATCAAGAAGAT; R: CTCCGGTAATGTCTCTGGG). The reaction system and reaction procedure are shown in Supplementary Tables 3, 4.

## Results

### Quantitative Trait Locus Primary Mapping

**FIGURE 2:**
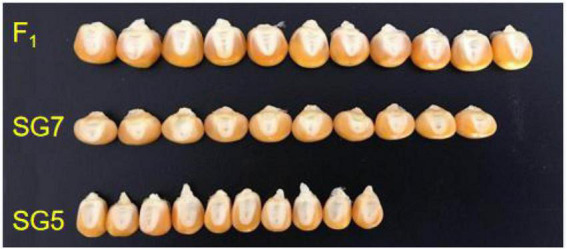
10 kernel width (KW) difference between SG5, SG7, and F_1_ hybrid seed derived from the cross between SG5 and SG7.

**FIGURE 3:**
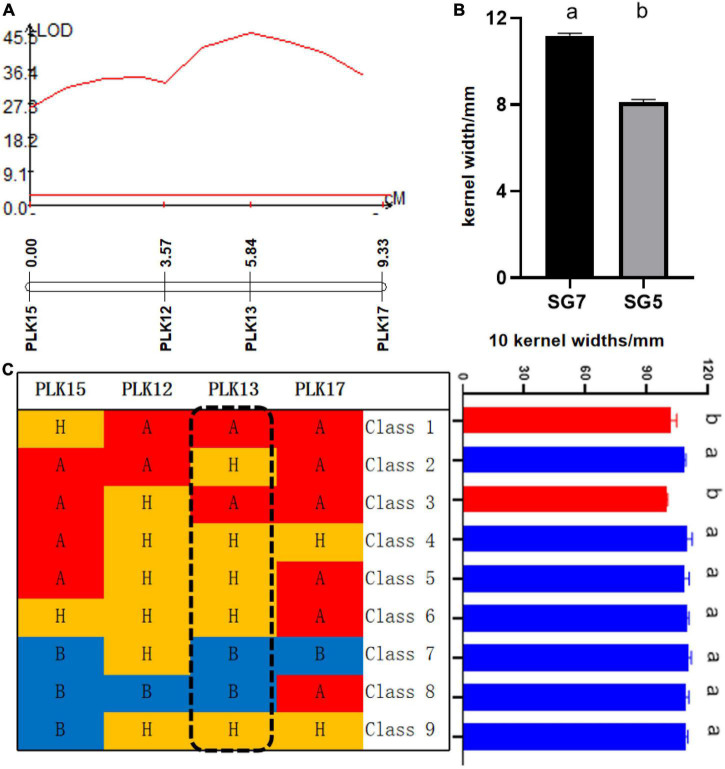
**(A)** Logarithm of the odds (LOD) profile of *qKW-1*, which was identified in the BC_4_F_2_ population. **(B)** Kernel width (KW) significance test of difference between SG5 and SG7, different letters a and b mean that difference was significant between SG5 and SG7. **(C)** *qKW-1* was mapped to a 5.76-cM genetic interval between the markers PLK12 and PLK17 by using 469 BC_4_F_2_ plants. Class 1–9 are shown for the recombinant plants with different recombination types. Graphical genotypes of recombinant lines and their KWs separated in BC_4_F_2_. Capital letter A with red color background represents the chromosomal segments for the homozygous SG5. Capital letter B with blue color background represents the chromosomal segments for the homozygous SG7. Capital letter H with yellow color background represents the chromosomal segments for the heterozygous alleles. Different lower case letters a and b indicate significant difference at 0.05 level.

The mean value of KW for the female parent SG5 was 8.07 mm, whereas for the male parent SG7 was 11.17 mm. The KW trait was highly and significantly different between SG5 and SG7 (Figures 2, 3B). A major QTL, *qKW-1*, for the KW trait was repeatedly detected in all three environments, with an average phenotypic variation contribution rate of 15.8%, by using the F_2_ and F_2:3_ populations developed from a cross between the maize inbred lines SG5 and SG7 (Wang et al., 2020). NILs will be developed for validating and fine mapping *qKW-1* loci next.

### Development of Near-Isogenic Lines and Phenotypic Evaluation

**FIGURE 4:**
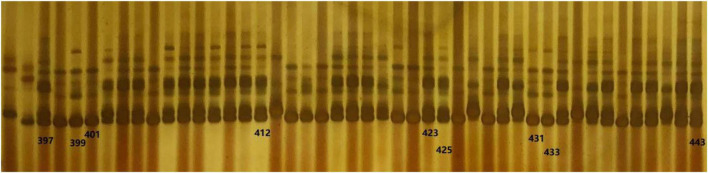
Part of genotype of simple sequence repeat (SSR) primer PLK13 on chromosome 3 in BC_4_C_2_ population. Lane 1 denotes genotype of female parent SG5, whereas lane 2 denotes genotype of recurrent parent SG7.

Four pairs of polymorphic SSR markers (Table 1) out of 20 SSRs (Supplementary Table 1) between donor parent SG5 and recurrent parent SG7 were obtained for marker-assisted selection from the BC_2_F_1_ to BC_4_F_2_ generation. The four markers PLK12, PLK13, PLK15, and PLK17 were found to be located at 31.55, 31.65, 32.51, and 33.89 Mb, respectively, on chromosome 3 by blasting maize B73 RefGen_v5. A total of 5 individuals with heterozygous genotypes for the *qKW-1* locus were selected from 103 BC_2_F_1_ plants by using the above four polymorphic SSR markers. The seeds of the 5 selected BC_2_F_1_ individuals were planted and subjected to continuous backcrossing with SG7. Plants with four heterozygous markers around the *qKW-1* locus on chromosome 3 were detected and selected in each generation from BC_2_ to BC_4_. In BC_4_F_1_, the plants heterozygous for four markers were selected for developing BC_4_F_2_. A total of 469 BC_4_F_2_ plants were obtained and planted in Qingdao for phenotyping KW traits in the summer of 2021. Figure 4 shows some of the segregation genotypes of SSR primers for PLK13 on chromosome 3 in the BC_4_F_2_ population.

**TABLE 1:** List of primers used for developing secondary genetic linkage map and fine mapping *qKW-1*.

Name	Forward	Reverse
PLK12	5′-GCAGCTAACGGTTTGATGCA-3′	5′-TTGCGTCGTTGCTTGAACAG-3′
PLK13	5′-GCGGATTGTTTTCGTCTCCG-3′	5′-TGATGCAAAGACACGAGCCA-3′
PLK15	5′-TGCACATTTACCTTCCACTGGA-3′	5′-GTGCAAGCGAGTCTTTTGGG-3′
PLK17	5′-CGAATCGCCGAAGACGTACA-3′	5′-TAGCAGTCGACGAACGGAAC-3′

*The four markers were located at 31.55, 31.65, 32.51, and 33.89 Mb on chromosome 3 by blasting maize B73 RefGen_v5.*

**FIGURE 5:**
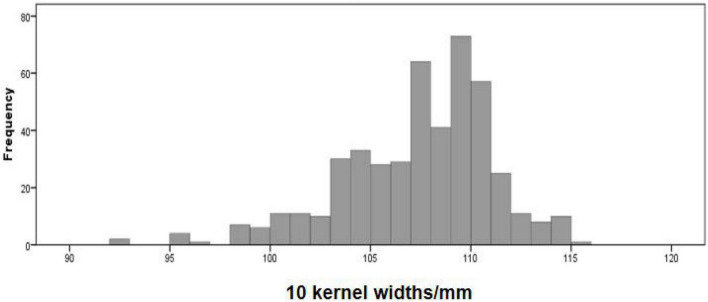
Distribution of kernel width (KW) traits in BC_4_F_2_ population derived from the cross of SG5 and SG7. SG5 as donor parent, whereas SG7 as recurrent parent.

**FIGURE 6:**
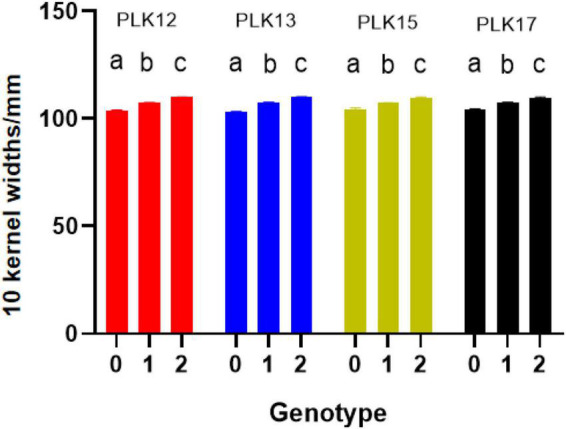
Phenotype significance test of difference for BC_4_F_2_ individuals (different letters indicate significant difference at the 0.05 level; 0 means maternal SG5 type, 1 means heterozygous type, 2 means paternal SG7 type).

The phenotype statistical analysis indicated that the frequency distribution of 10 KW s among BC_4_F_2_ lines exhibited a mixed distribution with transgressive segregation [range, 92.67–115.44 mm; mean, 107.27 mm; SG5, 80.7 mm; SG7, 111.7 mm (Figure 5); standard deviation, 0.18]. It showed significant genetic variation among the individuals. Statistical analysis of the phenotype of the BC_4_F_2_ population was based on the different segregating genotypes of the four pairs of markers. The results showed that if the marker genotype was the maternal type (0-band type), the 10 KWs were less than 104.49 mm, whereas if the marker genotype was the paternal type (2-band type), the 10 KWs were greater than 109.79 mm. The maternal genotype had a minimizing effect on KW, whereas the paternal genotype had an enlarging effect on KW (Table 2). To further compare the significance differences of the phenotypes with different segregating genotypes of the four pairs of markers, the BC_4_F_2_ population phenotypes were grouped according to different segregating genotypes. Variance analysis was performed. The analysis of variance (ANOVA) results of the four pairs of primers showed that the obtained *p*-values were all less than 0.01, reaching a very significant level. Among the four pairs of markers, the PLK13 marker had the largest *F*-value of 131.72, suggesting that the *qKW-1* locus had the strongest correlation or the closest genetic distance to PLK13 (Table 3). The multiple comparison results showed that the phenotypic differences of each of the four pairs of markers reached a significant level (Table 4 and Figure 6).

**TABLE 2:** Descriptive statistical analysis the phenotype of the BC_4_F_2_ population (0 means female SG5 type, 1 means heterozygous type, 2 means paternal SG7 type).

	Number^a^	Mean	SD^b^	SE^c^	95% CI of mean		Min	Max
					LCL^d^	UCL^e^		
**PLK12**								
0	84	103.58	3.93	0.43	102.73	104.43	92.83	112.77
1	273	107.40	3.42	0.21	107.00	107.81	92.67	115.44
2	104	109.89	2.12	0.21	109.48	110.30	104.36	114.69
**PLK13**								
0	86	102.91	3.77	0.41	102.10	103.72	92.67	110.73
1	264	107.54	3.17	0.20	107.16	107.93	98.18	115.44
2	112	109.98	2.04	0.19	109.60	110.36	104.36	114.69
**PLK15**								
0	100	104.49	4.22	0.42	103.65	105.32	92.83	115.44
1	247	107.22	3.50	0.22	106.78	107.65	92.67	114.72
2	113	109.84	2.08	0.20	109.45	110.23	104.11	114.69
**PLK17**								
0	105	104.11	4.37	0.43	103.27	104.96	92.67	114.72
1	244	107.53	3.22	0.21	107.12	107.94	98.18	115.44
2	107	109.79	2.19	0.21	109.37	110.21	103.88	114.69

*^a^Number of observations.*

*^b^Standard deviation.*

*^c^Standard error.*

*^d^Lower confidence limit.*

*^e^Upper confidence limit.*

**TABLE 3:** The results of phenotypic analysis of variance (ANOVA) of BC_4_F_2_ population grouped according to different marker genotypes (the first group for the maternal SG5 type, the second group for the heterozygous genotype, and the third group for the paternal SG7 type).

	Square sum	*df*	Mean square	*F* value	*P*-value
**PLK12**					
Between groups	1860.475	2	930.24	86.52	0.000
Within groups	4924.219	458	10.75		
**PLK13**					
Between groups	2476.775	2	1238.39	131.72	0.000
Within groups	4315.315	459	9.40		
**PLK15**					
Between groups	1521.836	2	760.92	66.16	0.000
Within groups	5255.889	457	11.50		
**PLK17**					
Between groups	1740.462	2	870.23	78.72	0.000
Within groups	5007.903	453	11.06		

**TABLE 4:** Difference significance analysis of phenotypes of BC_4_F_2_ populations (0 means maternal SG5 type, 1 means heterozygous type, 2 means paternal SG7 type).

	PLK12		PLK13		PLK15		PLK17	
G	Number^a^	Mean	Number	Mean	Number	Mean	Number	Mean
0	84	103.58 a	86	102.91 a	100	104.49 a	105	104.11 a
1	273	107.40 b	264	107.54 b	247	107.22 b	244	107.53 b
2	104	109.89 c	112	109.98 c	113	109.84 c	107	109.79 c

*^a^Number of observations.*

*Different letters a, b, and c indicates significant difference at the 0.05 level.*

### Near-Isogenic Population Background Detection

**FIGURE 7:**
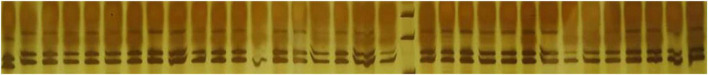
Part of genotype of simple sequence repeat (SSR) primers P17 on chromosome 7 in BC_4_C_2_ population. Lane 1 denotes genotype of female parent SG5, whereas lane 2 denotes genotype of recurrent parent SG7.

Theoretically, after a four-generation continuous backcross process, approximately 96.9% of the loci of the whole genome became homozygous in the BC_4_F_2_ population. To confirm that the genetic background of the BC_4_F_2_ population was homozygous, 45 randomly selected SSR markers from all 9 chromosomes except 3 were used for further genetic background analysis. The results showed that the genotypes of all 45 SSR markers were homozygous and were confirmed to be the same as that of the recurrent parent SG7. Figure 7 displays some of the genotypes of SSR marker P17 on chromosome 7, and all offspring genotypes are the same as SG7.

### Secondary Genetic Linkage Map Construction and Quantitative Trait Locus Analysis

From 2017 to 2019, a BC_4_F_2_ NIL population consisting of 469 lines was developed by introgressing the *qKW-1* genomic region of SG5 into the SG7 genetic background. A secondary linkage map with four markers (Table 1) around *qKW-1* was generated. The secondary linkage map was 9.33 cM (2.34 Mb) in length, and the genetic distances between each two adjacent markers were 3.57, 2.27, 0.80, 5.67, and 3.49 cM (Figure 3A). The order of markers on the genetic map is slightly different from that of the physical map. The order of the four markers on the genetic map was PLK15, PLK12, PLK13, and PLK17. By blasting maize B73 RefGen_v5, the four markers PLK12, PLK13, PLK15, and PLK17 were found to be located at 31.55, 31.65, 32.51, and 33.89 Mb, respectively, on chromosome 3. Then, the major QTL *qKW-1* was detected with the secondary linkage map of NILs by the CIM method in QTL Cartographer v2.5. *qKW-1* had an additive effect of 2.87 mm and explained 23.8% of the phenotypic variation. The LOD peak indicated that *qKW-1* was most likely located between PLK12 and PLK13, and the LOD value was 45.5 (Figure 3A). The distances from *qKW-1* to PLK12 and PLK13 were 2.23 and 0.04 cM, respectively, with a 95% confidence interval of 3.6–8.9 cM, which indicates a confidence distance of 5.3 cM.

To confirm the narrowed *qKW-1* interval, nine recombinant types, namely, Class 1–Class 9, were selected from 469 NILs. Class 1 indicates 5 recombinants with PLK12, PLK13, and PLK17 homozygous for SG5 and heterozygous for PLK15. Class 2 indicates 5 recombinants with PLK12, PLK15, and PLK17 homozygous for SG5 and heterozygous for PLK17. Class 3 indicates four recombinants with PLK13, PLK15, and PLK17 homozygous for SG5 and heterozygous for PLK12. Class 4 indicates 10 recombinants heterozygous for PLK12, PLK13, and PLK17 and PLK15 homozygous for SG5. Class 5 indicates 4 recombinants heterozygous for PLK12 and PLK13 and PLK15 and PLK17 homozygous for SG5. Class 6 indicates 4 recombinants heterozygous for PLK12, PLK13, and PLK15, whereas PLK17 is homozygous for SG5. Class 7 indicates 9 recombinants with PLK13, PLK15, and PLK17 homozygous for SG7, whereas PLK12 is heterozygous. Class 8 indicates 4 recombinants with PLK12, PLK13, and PLK15 homozygous for SG7, whereas PLK17 is heterozygous. Class 9 indicates 3 recombinants heterozygous for PLK12, PLK13, and PLK17, whereas PLK12 is homozygous for SG7. At PLK13 loci, Classes 1 and 3 were homozygous for SG5, whereas Classes 7 and 8 were homozygous for SG7 (Table 5). There was a significant difference in phenotypic values between the two sets of recombinant classes 1 and 3 and classes 7 and 8 (Figure 3C). The progeny test of homozygous segregants indicated that *qKW-1* was most likely located near PLK13. The selected overlapping recombinant chromosomes also supported the location of *qKW-1* mapped in BC_4_F_2_.

**TABLE 5:** Statistical analysis of phenotypic values from different kinds of recombinant types of near-isogenic lines (NILs) around the *qKW-1* region.

	No. of recombinants	10 kernels length/mm
Class 1^a^	5	101.60 ± 1.46
Class 2^b^	5	108.37 ± 0.38
Class 3^c^	4	99.93 ± 0.86
Class 4^d^	10	109.71 ± 0.86
Class 5^e^	4	108.35 ± 1.33
Class 6^f^	4	109.69 ± 0.69
Class 7^g^	9	110.74 ± 0.43
Class 8^h^	4	109.39 ± 0.66
Class 9^i^	3	109.56 ± 0.39

*^a^Class 1 indicates that 5 recombinants with PLK12, PLK13, and PLK17 Homozygous to SG5, whereas PLK15 heterozygous.*

*^b^Class 2 indicates 5 recombinants with PLK12, PLK15, and PLK17 homozygous to SG5, whereas PLK17 heterozygous.*

*^c^Class 3 indicates four recombinants with PLK13, PLK15, and PLK17 homozygous to SG5 whereas PLK12 heterozygous.*

*^d^Class 4 indicates 10 recombinants with PLK12, PLK13, and PLK17 heterozygous whereas PLK15 homozygous to SG5.*

*^e^Class 5 indicates 4 recombinants with PLK12 and PLK13 heterozygous whereas PLK15 and PLK17 homozygous to SG5.*

*^f^Class 6 indicates 4 recombinants with PLK12, PLK13, and PLK15 heterozygous whereas PLK17 homozygous to SG5.*

*^g^Class 7 indicates 9 recombinants with PLK13, PLK15, and PLK17 homozygous to SG7, whereas PLK12 heterozygous.*

*^h^Class 8 indicates 4 recombinants with PLK12, PLK13, and PLK15 homozygous to SG7, whereas PLK17 heterozygous.*

*^i^Class 9 indicates 3 recombinants with PLK12, PLK13, and PLK17 heterozygous, whereas PLK12 homozygous to SG7.*

### Candidate Gene Prediction

The RNA-seq procedure was conducted for 18 RNA grain samples at different developmental stages. According to the above results, the 95% confidence interval of *qKW-1* was 3.6–8.9 cM, which was located in the PLK12 to PLK17 marker interval. The physical distance between PLK12 and PLK17 was 2.34 Mb. The 2.34 Mb physical interval of *qKW-1* encompassed 45 protein coding genes (Supplementary Table 2). After DEG analysis, a total of 12 protein-coding genes were differentially expressed and retained in the *qKW-1* physical intervals (Table 6). Previous studies indicated that non-specific phospholipase C (NPC) is involved in plant growth, development, and stress responses (Yang et al., 2021). *NRT1.1B* is associated with root microbiota composition and nitrogen use in field-grown rice (Zhang et al., 2019). GRMZM2G083176 encodes NPC, and GRMZM2G081719 encodes the nitrate transporter 1 (NRT1) protein. The two genes GRMZM2G083176 and GRMZM2G081719 were predicted to be candidate genes of *qKW-1*, which is most likely responsible for *qKW-1*.

**TABLE 6:** Differentially expressed genes (DEGs) out of 40 protein coding genes in 2.34-Mb physical interval on chromosome 9 and candidate gene predicted for *qKW-1*.

Gene ID (B73 RefGen_v3)	Start (bp)	End (bp)	Length	Annotation	LogFC^a^ or RCP1/RCP2^b^		
					Day 5^c^	Day 10	Day 15
GRMZM2G083176	32125258	32128214	2698	Protein NRT1	–1.56^d^	–1.51	–1.60
GRMZM2G069827	32231832	32240883	1045	Charged multivesicular body protein 5	0.59	0.38	0.41
GRMZM2G070167	32241094	32242303	980	Light-regulated protein	1.18	2.23	1.11
GRMZM2G081719	32410415	32412583	1971	Non-specific phospholipase C6	–0.61	–1.58	–0.81
GRMZM2G339866	32467009	32467818	810		–1.06	–0.11	1.38
GRMZM2G099960	32534481	32535195	715		–0.27	0.58	1.46
GRMZM2G451975	32720982	32722838	1857		–1.35	–2.26	–0.59
GRMZM2G470981	32783855	32792053	1266	RS8_MAIZE 40S ribosomal protein S8	25.21/0	55.21/0	39.99/0
GRMZM2G132936	32949193	32958127	3399	Deleted in azoospermia protein 2	–0.85	0.10	0.07
GRMZM2G058896	33157785	33163820	383		5.75	6.70	5.86
GRMZM2G328893	33565965	33571557	1817	Cysteine synthase, chloroplastic/chromoplastic	–1.21	–1.77	–2.68
GRMZM2G134182	33763992	33765594	1243		1.76	2.48	2.95

*^a^Log 2 ratio, number of folds the gene is differentially expressed in RNA-seq.*

*^b^Different of readcounts between P_1_ and P_2_.*

*^c^Day 5, Day 10, and Day 15 indicate grain samples collected after selfing 5, 10, and 15 days between the two parents SG5 and SG7.*

*^d^Positive sign indicates gene transcript expressed high in SG5, whereas negative sign indicates gene transcript expressed high in SG7.*

### Reverse Transcription-Polymerase Chain Reaction Analysis

To verify the accuracy of the RNA-seq results, the actin gene was used as a reference gene, and two differentially expressed candidate genes, GRMZM2G083176 and GRMZM2G081719, were used for RT-qPCR analysis. The results showed that the change trend of the expression levels of the two candidate genes was basically consistent with the change trend of the gene expression levels obtained from RNA-seq analysis. The expression trends of GRMZM2G083176 and GRMZM2G081719 are basically consistent. There was no significant difference in the expression levels of SG5 and SG7 between the two stages on Day 0 (grain samples collected before selfing for 10 days) and Day 30 (grain samples collected after selfing for 30 days). At Day 5, Day 10, and Day 15 (grain samples collected after selfing for 5, 10, and 15 days between the two parents SG5 and SG7), the expression levels of the two candidate genes in SG7 were significantly increased and significantly higher than those in SG5. The expression level of GRMZM2G083176 reached the highest level on Day 10, whereas the expression level of GRMZM2G081719 reached the highest level on Day 5. The expression levels of the two genes were significantly different during grain development stages. RT-qPCR analysis results further verified the correctness of GRMZM2G083176 and GRMZM2G081719 prediction as candidate genes.

**FIGURE 8:**
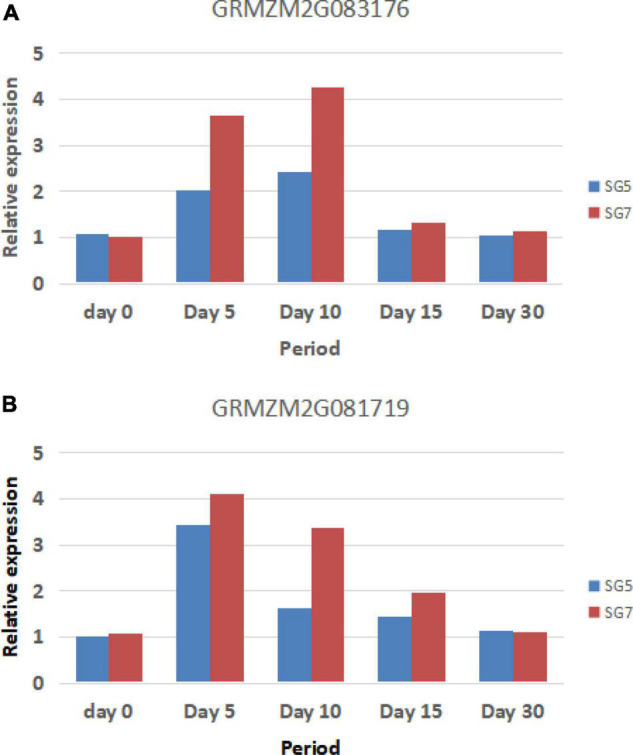
The reverse transcription-polymerase chain reaction (RT-PCR) validation genes and their expression levels. Panel **A** indicates GRMZM2G083176 expression levels, whereas panel **B** indicates GRMZM2G081719 expression levels (Day 0 indicates grain samples collected before selfing 10 days, whereas Day 5, Day 10, Day 15, and Day 30 indicate grain samples collected after selfing 5, 10, 15, and 30 days between the two parents SG5 and SG7).

Further studies are still needed to clarify whether the two genes regulate grain development independently or interact to affect grain development (Figure 8).

## Discussion

Due to different genetic materials and genetic backgrounds, the results obtained by different researchers are also different. Liu (2013) identified 16 QTLs related to KW, mainly distributed on chromosomes 1, 2, 3, 4, 5, and 9. Li et al. (2009) identified a KW–related QTL located on chromosome 8 in a single ecological environment and concluded that the KW trait significantly affected the seed setting percentage. Qin et al. (2015) mapped the KW QTL qkwid10 on chromosome 10. Chen et al. (2017) conducted meta-QTL analysis based on collecting information on QTLs conferring maize yield-related traits from 33 published studies. A total of 76 MQTLs for maize yield and its related traits were identified on all 10 maize chromosomes, with the number per chromosome ranging from four on chromosome 4–10 on chromosome 5 (Chen et al., 2017). According to meta-QTL analysis results, MQTL-19 for ear-related traits and kernel-related traits was found to be limited within a physical interval of 31.06–63.84 Mb by blasting maize B73 RefGen_v4. The physical interval of *qKW-1* in this study overlapped with the MQTL-19 interval, but with more decreased physical intervals. The genetic effect of the *qKW-1* region is relatively stable under different genetic backgrounds, and it can be used as an important candidate region for marker-assisted breeding and map-based cloning in maize.

Near-isogenic lines can be developed by backcrossing, selecting residual heterozygous lines in RILs, and using mutants. The genetic background of NILs is the same, with only the target trait gene differing. NILs are an ideal material for genetic research at the gene level. Molecular markers closely linked to the target gene can be accurately screened by using NILs, which is convenient for fine mapping of the target gene. For example, the early tomato anti-virus gene *Tm-2a* (Young et al., 1988) and the semidwarf stem gene *sdg* (Liang et al., 1994) of rice were identified by using NILs. Using NILs to analyze gene functions can eliminate interference from other genes and genetic backgrounds. In addition, NILs are more helpful for accurately analyzing the expression differences of target genes. For example, Zhao et al. (2011) introduced the exogenous pea ferritin gene (*Pea-Fer*) into Xiushui 11 rice and constructed NILs. The results showed that the *Pea-Fer* gene could significantly increase the iron content in rice grains (Zhao et al., 2011). NILs can also be directly used as breeding materials. For example, the multiline varieties used for rice blast resistance in Japan in the early days are actually a group of NILs containing different rice blast resistance genes (Koizumi, 2001). The development of NILs by using only backcross technology requires much work and time. In this study, the method of developing NILs using backcrossing combined with molecular marker-assisted selection greatly accelerated the development of NILs.

A secondary genetic linkage map was constructed by using the advanced genetic population developed in this study. The target *qKW-1* was limited in a 2.34-Mb physical interval. In order to avoid the omission of important genetic information, the range of candidate genes was defined according to the marker interval where the confidence interval located in. The confidence interval of *qKW-1* in this study was 3.6–8.9 cM, whereas the marker interval PLK12–PLK17 was 3.6–9.3 cM in the secondary genetic linkage map; therefore, the 2.34-Mb physical interval between PLK12 and PLK17 was applied for candidate gene analysis. Since the 2.34-Mb interval was not small enough and unknown genetic elements, RNA-seq and RT-qPCR experiments were conducted to analyze DEGs between the two parents SG5 and SG7. Finally, 12 DEGs were obtained, and the 12 genes were used for comparative genomics studies. Previous studies indicated that non-specific phospholipase C is involved in plant growth, development and stress responses (Yang et al., 2021). Results from Cao et al. (2016) indicate that the overexpression of NPC1 leads to brittle stem nodes and panicle nodes and increased seed shattering due to a decrease in mechanical strength in nodes tissues. *NRT1.1B* is associated with root microbiota composition and nitrogen use in field-grown rice (Zhang et al., 2019). Hu B. et al. (2015) used the IR24 chromosome single-segment substituted lines of Nipponbare background to map QTLs related to the ClO_3_^–^ resistance and confirmed that the QTL is nitrate transporter gene OsNRT1.1B through map-based cloning technology. The results showed that indica OsNRT1.1B has higher NO_3_^–^ absorption and transport activities than japonica OsNRT1.1B. Multisite field experiments indicated that indica OsNRT1.1B can significantly improve the yield and nitrogen use efficiency (NUE) of japonica rice varieties, which has important application value (Hu B. et al., 2015). GRMZM2G083176 encodes NPC and GRMZM2G081719 encodes NRT1 protein. The two genes GRMZM2G083176 and GRMZM2G081719 were predicted as a candidate gene of *qKW-1*, which is most likely responsible for *qKW-1*. The obtained results not only lay a foundation for further *qKW-1* marker-assisted breeding but also provide ideal materials for map-based cloning and molecular mechanism analysis of the *qKW-1* gene in maize.

## Conclusion

The major QTL *qKW-1* for KW was verified and fine-mapped between the PLK12 and PLK13 marker intervals, with a distance of 2.23 cM to PLK12 and 0.04 cM to PLK13, a confidence interval of 5.3 cM and a phenotypic contribution rate of 23.8%. The QTL mapping result obtained was further validated by using selected overlapping recombinant chromosomes on the target segment of maize chromosome 3. Transcriptome analysis and RT-qPCR validation were conducted, and the results provide further proof of the two candidate genes GRMZM2G083176 and GRMZM2G081719, which are most likely responsible for *qKW-1*.

## Data Availability Statement

The RNA-seq data presented in this study are deposited in the NCBI SRA repository, accession number PRJNA673992.

## Author Contributions

YZ and XM developed F_2_, F_2:3_, and BC_4_F_2_ population and genotyping. MZ, JW, and GW involved in phenotypes investigation. CS analyzed the data and drafted the manuscript. All authors read and approved the final manuscript.

## Conflict of Interest

The authors declare that the research was conducted in the absence of any commercial or financial relationships that could be construed as a potential conflict of interest.

## Publisher’s Note

All claims expressed in this article are solely those of the authors and do not necessarily represent those of their affiliated organizations, or those of the publisher, the editors and the reviewers. Any product that may be evaluated in this article, or claim that may be made by its manufacturer, is not guaranteed or endorsed by the publisher.

## Footnotes

1
ftp://ftp.ensemblgenomes.org/pub/plants/release-29/fasta/zea_mays/dna/Zea_mays.AGPv3.29.dna.toplevel.fa.gz

2
https://www.ncbi.nlm.nih.gov/tools/primer-blast/
