# Supplementary material for: Validating a Major Quantitative Trait Locus and Predicting Candidate Genes Associated With Kernel Width Through QTL Mapping and RNA-Sequencing Technology Using Near-Isogenic Lines in Maize
Source: Front Plant Sci. 2022 Jun 30;13:935654. doi: 10.3389/fpls.2022.935654 (PMC9280665; doi:10.3389/fpls.2022.935654)
Supplement: Supplementary file 1 [file Table_1.DOC]

**Table 3.** 10 μl qRT-PCR system

component part volume (μl)

2X SYBR® Green *Pro Taq* HS Premix 5 μl

Template 1 μl

Primer F (10μM) 0.2 μl

Primer R (10μM) 0.2 μl

ROX Reference Dye (4μM) *^3,4^ 0.2 μl

RNase free water Up to 10 μl

**Table 4.** qRT-PCR reaction conditions

| Step | Temperature | time | cycles |
| --- | --- | --- | --- |
| Step 1 | 95℃ | 30s |  |
| Step2 | 95℃ | 5s |  |
| Step 3 | 58℃ | 30s | 40 |
|  | 72℃ | 30s |  |
| Step 4 | 95℃ | 15s |  |
| Step 5 | 60℃ | 60s |  |
|  | 95℃ | 15s |  |
