# Supplementary material for: Validating a Major Quantitative Trait Locus and Predicting Candidate Genes Associated With Kernel Width Through QTL Mapping and RNA-Sequencing Technology Using Near-Isogenic Lines in Maize
Source: Front Plant Sci. 2022 Jun 30;13:935654. doi: 10.3389/fpls.2022.935654 (PMC9280665; doi:10.3389/fpls.2022.935654)
Supplement: Supplementary file 3 [file Table_3.DOCX]

**Table 1.** List of primers used for polymorphism screening in a 8.65 Mb physical interval which qKW-1 located in.

| Name | Forward | Reverse |
| --- | --- | --- |
| PLK1 | 5’-AGCAAGGGAGAGGGAGAGAG | 5’-TCGTTTGCAAACAACGCTGT |
| PLK2 | 5’-GTGGGCTATGTATCCGAGGC | 5’-CTGCGACATGGATGGAGAGG |
| PLK3 | 5’-GATCATTTGGTCGCCGAAGC | 5’-CCTCGCATGACCGTAGACTC |
| PLK4 | 5’-TTTCTACGGCGAGTACACGG | 5’-TGAGGATGAATTTGCCACTAGA |
| PLK5 | 5’-GACAAGGAGATGGGTAGGCG | 5’-GCGAGATGGACACAAGGGAA |
| PLK6 | 5’-TCTATCTGGCCAGCAAGTGC | 5’-ACATCAACACAGGTTTGCTGG |
| PLK7 | 5’-ACACCCTTAATCTGTAGCATGCA | 5’-GGCTTGCACATGCTACATCG |
| PLK8 | 5’-CCTACACTAGGGTGGGCTTG | 5’-TCATAAAGGGGCATCTCGGC |
| PLK9 | 5’-ATAATGGCGCGCAGTTCTCC | 5’-TGTGCCACATCTGACAGCAA |
| PLK10 | 5’-TGTTTTAGAATGGCTGCAGTGT | 5’-TGACCAGTCTACAATGCACATCT |
| PLK11 | 5’-TTATGAACACCTCCCCCAGC | 5’-CGCGGAAGAGTGGTGCTATT |
| PLK12 | 5’-GCAGCTAACGGTTTGATGCA-3’ | 5’-TTGCGTCGTTGCTTGAACAG-3’ |
| PLK13 | 5’-GCGGATTGTTTTCGTCTCCG-3’ | 5’-TGATGCAAAGACACGAGCCA-3’ |
| PLK14 | 5’-CGTATGCATGTAGACACCCGA | 5’-CGACGCACACTTGTCACTTG |
| PLK15 | 5’-TGCACATTTACCTTCCACTGGA-3’ | 5’-GTGCAAGCGAGTCTTTTGGG-3’ |
| PLK16 | 5’-GACAAACCGTGAGTGGCCTA | 5’-CGCCCGATCACTAAGCTCTC |
| PLK17 | 5’-CGAATCGCCGAAGACGTACA-3’ | 5’-TAGCAGTCGACGAACGGAAC-3’ |
| PLK18 | 5’-GCCCCGATTAGCTTGCTACT | 5’-CACGCTCGTCTACTCTCGTC |
| PLK19 | 5’-TTGGTCTCGGAGAATTGGCC | 5’-GTATGTGTGGGCAGCTGACA |
| PLK20 | 5’-GCCATCCGTCTTGTGCATTC | 5’-CAAAACACGCGTGAGGTCTG |
